# A Misplaced Molar

**Published:** 1896-04

**Authors:** W. J. Martin

**Affiliations:** Kankakee, Ill.


### A MISPLACED MOLAR.

By W. J. MARTIN, V.S.,
KANKAKEE, ILL.

TIME, September, 1894; subject bay mare, seven years old, presenting a running sore at the base of right ear from time of being colt.

*Operation.* Mare was cast; the parts being washed, a round hard tumor could be felt at the antero-internal base of the ear. A straight incision of two inches in length was made over the tumor and carried down upon it; pressure of the fingers was then applied, and there came out a good representation of a molar tooth, somewhat necrosed around the fang portion.

This is the second instance met with in my practice of a similar condition in the same position; the first case in a young colt but a few weeks old, and was recorded in this JOURNAL of August, 1890. This latter one was adherent to the skull, and bone-forceps was used in extracting it. In both instances the flow of pus from the openings was copious.

